# Right Atrial Mass and Severe Symptomatic Anemia as the Initial Presentation of Renal Cell Carcinoma

**DOI:** 10.7759/cureus.64516

**Published:** 2024-07-14

**Authors:** Leslie-Joy Romero, Mona Ghias, Hugo Carducci, Nicholas Mains, Kevin Bogdansky

**Affiliations:** 1 Pediatric Hospital Medicine/Internal Medicine, West Virginia University, School of Medicine, Morgantown, USA; 2 Internal Medicine, West Virginia University, School of Medicine, Morgantown, USA; 3 Internal Medicine/Pediatrics, West Virginia University, School of Medicine, Morgantown, USA; 4 Nephrology, West Virginia University, School of Medicine, Morgantown, USA

**Keywords:** liver mass, right atrial cardiac mass, severe symptomatic anemia, metastatic renal cell carcinoma, renal cell carcinoma

## Abstract

We present a case of a 63-year-old male with a history significant for hypertension and a 45-pack-year smoking history who presented with severe symptomatic anemia. Rather quickly, upon imaging, he was found to have a 10 cm liver mass, a right renal mass, and a right atrial mass. A liver biopsy was performed and confirmed metastatic renal cell carcinoma (clear cell variant). Due to the extensive disease burden and patient preference, curative surgery was not pursued. This case highlights the rare but critical complications that can present as the initial presentation of renal cell carcinoma.

## Introduction

Renal cell carcinoma (RCC) is a rapidly progressive malignancy that accounts for approximately 3% of all adult malignancies, with kidney and renal pelvis cancers accounting for 4.1% of newly diagnosed adult cancers in the United States [1,2]. Due to the aggressive nature of this tumor and the high potential for metastases, RCC is usually discovered as an incidental finding on imaging, with 25% of patients having metastatic disease [3,4]. RCC with cardiac metastasis is a rare phenomenon, with extension into the inferior vena cava occurring in 15% of cases [3,4]. Extension into the right atrium typically occurs in 1% of the presenting cases [1,3,4]. This report presents a case of a patient with metastatic RCC presenting with symptomatic anemia, right atrial mass, and large hepatic metastases.

## Case presentation

The patient is a 63-year-old male with a medical history of hypertension and tobacco use disorder (45-pack-year smoking history) who presented initially for dyspnea on exertion and chest pressure. The patient complained of a steady decline in his health over one month, with exertional dyspnea limiting his activity. On presentation, initial lab analysis showed severe microcytic anemia with a hemoglobin of 3.5 g/dL (normal 13.8-17.2 g/dL). Iron studies were consistent with severe iron deficiency anemia with normal renal function (glomerular filtration rate greater than 90). 

**Figure 1:**
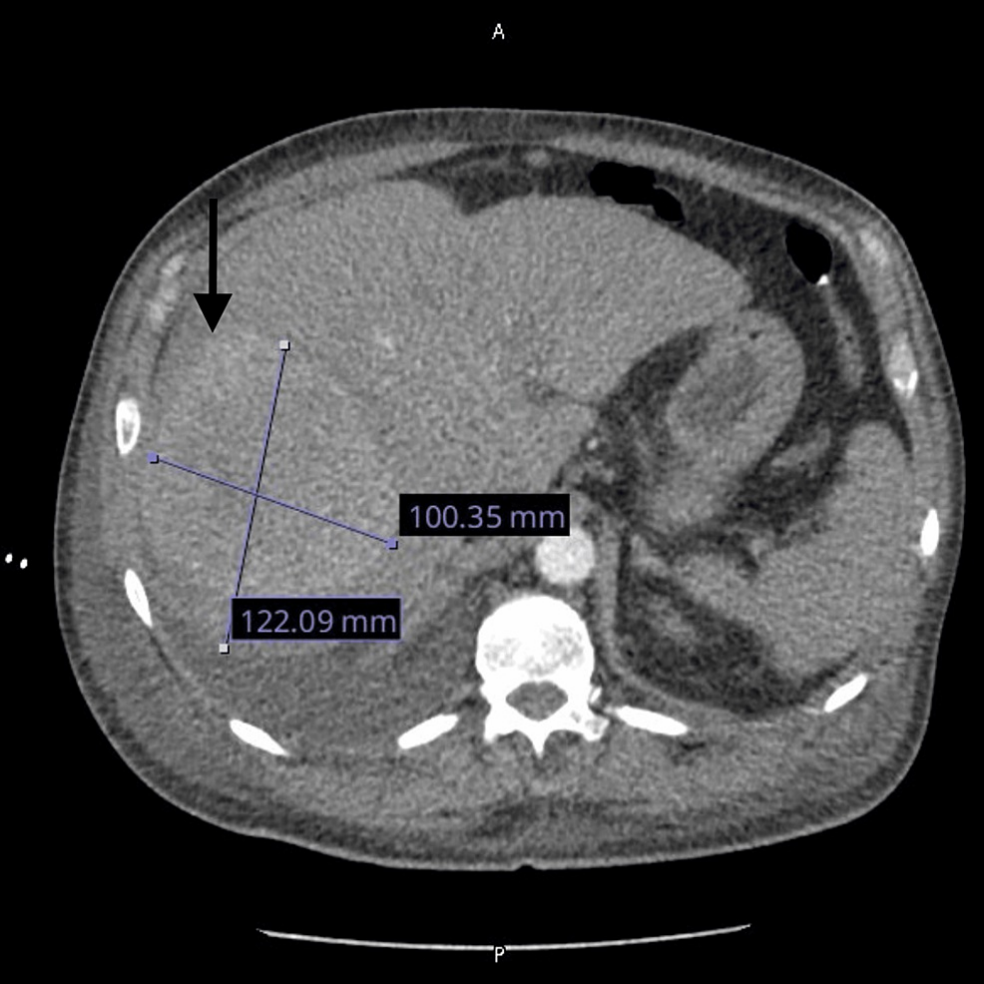
Hyperdense liver mass Computed tomography (CT) of 10.04 x 12.21 cm liver mass

**Figure 2:**
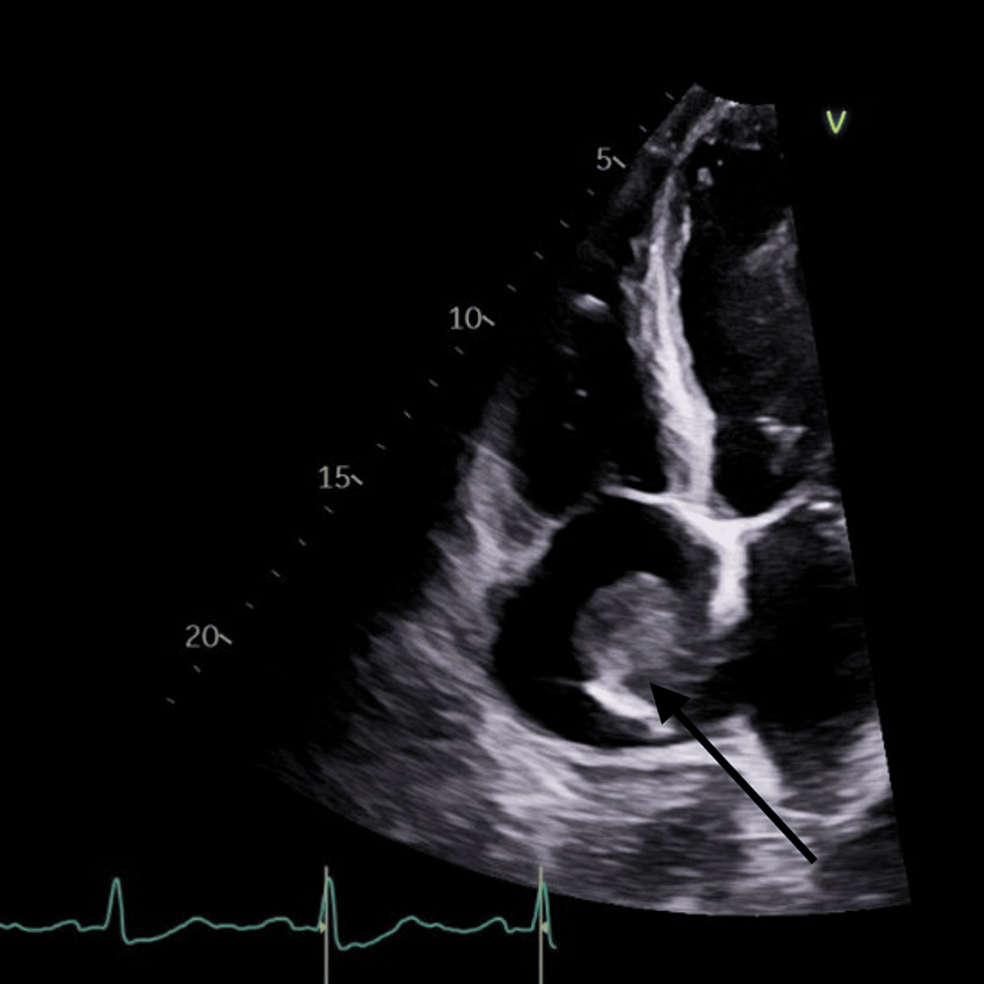
Right atrial mass Right atrial mass seen on a transthoracic echo

A chest X-ray showed cardiomegaly and a small right pleural effusion. Computed tomography (CT) imaging revealed a large (10.04 x 12.21 cm) hyperdense liver mass (Figure 1), multiple bilateral pulmonary nodules, and a possible right atrial mass (Figure 2).

CT of the abdomen and pelvis confirmed a large right renal mass with invasion of the adjacent bowel, a right atrial mass extending into the inferior vena cava, and extensive metastatic disease involving the liver, lungs, and lymph nodes.

A biopsy of the liver mass confirmed metastatic RCC, a likely clear cell variant, based on immunohistochemical staining. 

A transthoracic echocardiogram (TTE) revealed global hypokinesis of his left ventricle (LV) with an ejection fraction of 40%, grade II diastolic dysfunction, and a 3.2 x 4.2 cm right atrial mass, along with moderate pulmonary hypertension. 

Cardiac surgery was consulted and given the extensive metastatic disease and high-risk surgery, surgical intervention was not offered. The patient decided to pursue palliative care, focusing on symptom management.

The patient received multiple blood transfusions for symptomatic anemia, and his hemoglobin eventually stabilized. Anticoagulation for the right atrial mass was deferred due to the severe, persistent symptomatic anemia. He received iron supplementation and was discharged with close outpatient follow-up.

## Discussion

Renal cell carcinoma (RCC) typically presents as a triad of flank pain, hematuria, and renal mass [4]. However, this triad is typically uncommon, and most RCC is described as having various presentations due to the dissemination of disease to distant metastatic sites [1]. More than 70% of all renal cancers are found incidentally by imaging modalities done for other reasons [1]. Common sites of metastatic disease for RCC are the lungs, adrenal glands, brain, and most intra-abdominal organs [1]. RCC can invade local vasculature into the renal vein, where, in 15% of cases, it extends into the inferior vena cava [3,4]. Metastasis into the right atrium is typically rare and seen in about 1% of cases [1,3,4].

This case highlights the aggressive nature of metastatic RCC, which can present with diverse clinical manifestations. Symptomatic anemia due to bone marrow infiltration is a common complication. In this case, the patient also presented with a right atrial mass, a rare but potentially life-threatening complication of RCC. Due to the extensive metastatic disease and high-risk surgical intervention for treatment, the patient was too high-risk for surgical intervention. 

Treatment options for metastatic RCC depend on various factors, including tumor stage, performance status, and patient preference. A co-surgical approach would involve cardiopulmonary bypass with deep hypothermic circulatory arrest, involving both urologic and cardiac surgery teams [4,5]. The surgery involves a radical nephrectomy and cardioplegic cardiac and circulatory arrest. Afterward, a cavotomy, an atriotomy, and a subsequent thrombectomy are typically performed to remove the thrombus [6].

## Conclusions

This case report demonstrates the varied and aggressive clinical presentation of metastatic RCC, with a rare initial presentation of a large right atrial mass and symptomatic anemia. Early diagnosis and prompt treatment are crucial for improving patient outcomes. Multidisciplinary management, including medical oncology, urology, and cardiology, may be necessary depending on the specific presentation of the disease.
